# Model-Observer Similarity, Error Modeling and Social Learning in Rhesus Macaques

**DOI:** 10.1371/journal.pone.0089825

**Published:** 2014-02-24

**Authors:** Elisabetta Monfardini, Fadila Hadj-Bouziane, Martine Meunier

**Affiliations:** 1 INSERM, U1028, Lyon Neuroscience Research Center, ImpAct Team, Lyon, France; 2 CNRS, UMR5292, Lyon Neuroscience Research Center, ImpAct Team, Lyon, France; 3 University Lyon, Lyon, France; 4 Institut de Médecine Environnementale, Paris, France; Institut Pluridisciplinaire Hubert Curien, France

## Abstract

Monkeys readily learn to discriminate between rewarded and unrewarded items or actions by observing their conspecifics. However, they do not systematically learn from humans. Understanding what makes human-to-monkey transmission of knowledge work or fail could help identify mediators and moderators of social learning that operate regardless of language or culture, and transcend inter-species differences. Do monkeys fail to learn when human models show a behavior too dissimilar from the animals’ own, or when they show a faultless performance devoid of error? To address this question, six rhesus macaques trained to find which object within a pair concealed a food reward were successively tested with three models: a familiar conspecific, a ‘stimulus-enhancing’ human actively drawing the animal’s attention to one object of the pair without actually performing the task, and a ‘monkey-like’ human performing the task in the same way as the monkey model did. Reward was manipulated to ensure that all models showed equal proportions of errors and successes. The ‘monkey-like’ human model improved the animals’ subsequent object discrimination learning as much as a conspecific did, whereas the ‘stimulus-enhancing’ human model tended on the contrary to retard learning. Modeling errors rather than successes optimized learning from the monkey and ‘monkey-like’ models, while exacerbating the adverse effect of the ‘stimulus-enhancing’ model. These findings identify error modeling as a moderator of social learning in monkeys that amplifies the models’ influence, whether beneficial or detrimental. By contrast, model-observer similarity in behavior emerged as a mediator of social learning, that is, a prerequisite for a model to work in the first place. The latter finding suggests that, as preverbal infants, macaques need to perceive the model as ‘like-me’ and that, once this condition is fulfilled, any agent can become an effective model.

## Introduction

Much attention is currently devoted to the mechanisms that specify when individuals copy others and from whom they learn [1]–[4]. Reward-based learning, an essential life skill that allows to distinguish rewarded from unrewarded items or actions, could provide valuable insight into this issue. First, we know a lot about its behavioral determinants and neural underpinnings thanks to decades of research in neuroscience [5]–[6]. Second, it is a form of learning that is easily acquired socially via observation of the outcome of others’ choices. Monkeys, for example, have repeatedly been shown to learn novel stimulus-outcome associations faster after observation of a conspecific over a 50-year period [7], [8], and across a variety of reward-based learning skills: object discrimination [7]–[10], reward schedule [11], token exchange [12], ordinal sequence learning [13], and pattern-guided foraging [14]. Here, the aim was to understand why the same monkeys that reliably learn from conspecifics do not systematically learn from a human model [8], [12], [15]–[17]. The idea was that understanding what makes transmission of information from human to monkey successful provides a unique window into phylogenetically ancient mediators and moderators [18] of social learning that operate even in the absence of language, refined intelligence, or sophisticated culture, and that are capable of overruling species specificities.

Two studies reported failed human-to-monkey transmission of knowledge. In both of them, the human model was used to show that monkeys do not learn from ‘stimulus enhancement’, i.e. by the simple process of having their attention drawn towards the rewarded stimuli [19]. Brosnan and de Waal [12] used token exchange in brown capuchins; we used object discrimination in rhesus macaques [8]. In both cases, the (ineffective) human model showed only the correct object-reward or token-reward associations; no error was shown, and the rewards were never eaten. On the opposite, Genovesio and colleagues reported three instances of effective human modeling [15]–[17], including one [17] obtained using the very same token exchange paradigm as Brosnan and de Waal [12]. There, the (effective) human model presented all possible token-reward associations, the incorrect and the correct ones, and consumed the rewards as a monkey would do. Based on these findings, the present study questions whether human models fail when their behavior (seeking the animal’s attention, neglecting high-value food reward) strays too far away from the monkeys’ own behavior or, alternatively, when they present a faultless performance with only correct responses and no errors.

Cross-species social interactions and learning are possible in primates both behaviorally and neurally. Human infants can interact and learn from non-human agents such as a robot [20] or a puppet [21] provided they first saw the agent act in a social fashion that they perceive as a ‘like-me’ behavior, a behavior that resembles their own [22]. The brain is also equipped to detect “like-me-ness” in others species’ behavior. The monkey mirror neurons respond to humans’ goal-directed actions and the human mirror system is activated by an action such as biting a piece of food regardless of the agent performing it, a man, a monkey, or a dog [23], [24]. So, monkeys may have failed to learn from ‘stimulus-enhancing’ human models because they could not find a ‘like-me-ness’ in the model’s behavior. Indeed, these models ended up neglecting highly desirable food, a behavior that is not very rational, especially in despotic species such as rhesus macaques that, even in the mother-offspring context, are not keen on food-sharing [25]. Supporting this hypothesis is evidence that the more alike observers are to models, the better the social learning, in both human [26], [27] and non-human animals [28]–[29]). For examples, in human teenagers writing argumentative texts, weak learners learn best from weak models while good learners learn best from good models [30]; in chimpanzees [31] and capuchins [32], daughters copy their mothers more reliably than do sons.

The other factor that may have impeded knowledge transmission from ‘stimulus-enhancing’ humans to monkeys is the absence of error in the model’s demonstration. In individual learning, subjects from many taxa (humans, monkeys, cats, birds) required to choose between two alternatives, one good, one bad, learn poorly if they happen to err on their first choice [10], [33]–[37]. On the contrary, in social learning, although learning from others’ successes can and do occur, humans, monkeys, and birds, all draw the most substantial and reliable benefit from others’ errors [7], [10], [38]. The power of others’ errors extends beyond two-choice discriminations. For example, human toddlers learn how to use a tool more effectively when shown an unsuccessful action prior to the target action, than when shown only the correct target action [39]. It is therefore possible that ‘stimulus-enhancing’ human models would have been effective had they demonstrated the incorrect responses in addition to or instead of the correct ones.

Here, we asked whether human models fail when they show a behavior too dissimilar from the animals’ own, or when they show a faultless performance devoid of error. We tested two groups of three rhesus macaques each in the same object discrimination task as before [8] with three different models: a familiar conspecific and two human models. The first human model behaved as in [8]: after having actively drawn the observer’s attention, this ‘stimulus-enhancing’ model displaced one of the objects of the pair, showing the underneath reward or lack thereof. The second human model, the ‘monkey-like’ human simply performed the task as the conspecific model did, choosing an object without making any special effort to capture the observer’s attention, and eating the reward when one was earned. Each model made erroneous choices for half of the demonstrated pairs, and correct choices for the other half.

The results identified model-observer similarity in behavior as a mediator of social learning. The monkey and ‘monkey-like’ models that both displayed a behavior resembling the observer’s own had the same beneficial effect on subsequent object discrimination learning, whereas the irrational behavior of the ‘stimulus-enhancing’ model tended, on the contrary, to perturb the animals. Modeled errors acted as a mere moderator. Relative to modeled successes, they maximized the models’ effects, optimizing learning from the effective models and further impeding learning from the ineffective one.

## Materials and Methods

The paragraphs below provide a brief description of the subjects and procedures. A more detailed account is available in [10].

### Ethics

The study was carried out in strict accordance with Directive 2010/63/UE of the European Parliament and of the Council of 22 September 2010 on the protection of animals used for scientific purposes. In accordance with the French transposition texts of Directive 2010/63/UE, the project was authorized by the French Ministry for Higher Education and Research (project N° 20-12-0401-006). This authorization was based on the ethical evaluation of the Committee on the Ethics of Experiments in Animals (C2EA) CELYNE registered at the national level as C2EA number 42.

### Enrichment

The animals housing quarters matched or exceeded the minimal surface, height, and volume (2 m^2^, 1.8 m, and 3.6 m^3^) required by Directive 2010/63/EU for adult macaques. Three types of enrichment were used. First, on a daily basis, monkey chow and fruits were hidden in primate rubber toys, and bird seeds were scattered in the litter shavings so that the animals spend a good part of their day foraging. Second, enclosures were equipped with wood poles, hammocks, swings, etc. to diversify exploratory activities. Third, one among a set of temporary devices (a puzzle, a movie, a swimming pool, a mirror, etc.) was provided each day for an hour or so.

### Subjects

Two trios of captive-born rhesus macaques (*Macaca mulatta*) participated in the study, one composed of 4-year-old males, the other of 3-year-old females. The two groups had never been involved in any experiment before. The three individuals composing each trio had been raised together since birth (female group) or weaning (male group). Each group was tested in its usual living quarters. The male group lived in a large indoor/outdoor enclosure and was tested outdoors (see [8]). The female group was laboratory-housed and was tested indoors in the communicating individual cages they shared. During testing, all three group members were present, each in a separate compartment, the two members playing the observer and actor roles being placed either at a 90° angle (male group) or face to face (female group). All monkeys had visual access to the experiment, but only the actor could reach for the objects. Systematically keeping all trio members during testing precluded any social facilitation/inhibition, i.e. any benefit/impediment due to the mere presence of others [40], [41]. The animals had free access to water and received normal food rations of fresh fruits and monkey chow once a day after the testing session. The study capitalized on monkeys’ spontaneous willingness to monitor the behavior of social partners.

### Task

The object discrimination task consisted in presenting pairs of objects, each object covering a food well where chocolate candies could be hidden. For each pair, one of the two objects, always the same, was rewarded. Objects were toys, cardboard boxes, plastic containers, etc. widely varying in shape, size, texture, and color. Two types of pairs were mixed within each list in order to assess two different learning conditions within each and every session: six pairs were learned after having had the opportunity to observe a model (hereinafter referred to as ‘social pairs’ or social learning condition) whereas the remaining three pairs had to be learned purely individually without the help of prior observation of a model (hereinafter ‘individual pairs’ or individual learning condition). Each individual saw a different (9-pair) list each time he/she participated as actor or model.

### Procedure

**Figure 1:**
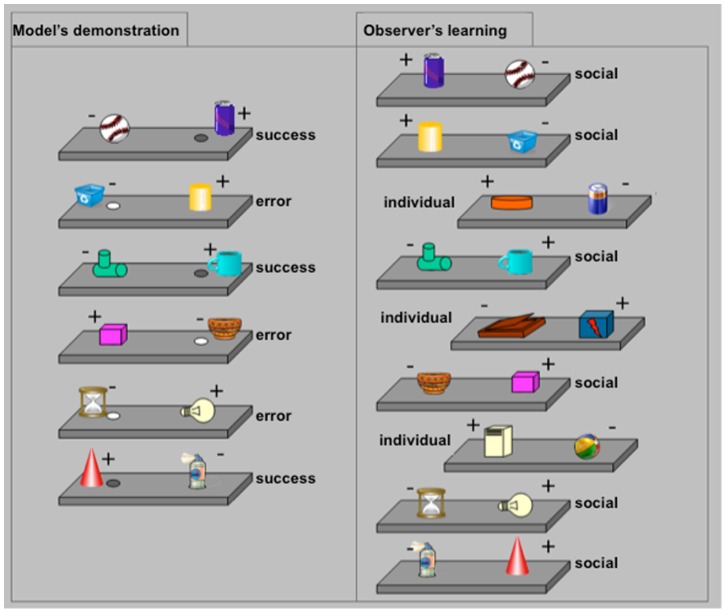
Schematic representation of a learning session. One of the three models first showed six pairs, the so-called ‘social pairs’, modeling the correct response (success) for three of them, and the incorrect response (error) for the other three. Then, the observer monkey was tested. Three additional pairs were inserted in the list (the ‘individual pairs’) and the now complete 9-pair list was presented 10 times to the monkey. The observer’s performance was thus evaluated over 10 hands-on trials for all pairs, whether ‘individual’ or ‘social’.

As illustrated in Figure 1, each session started with the model’s demonstration of the six ‘social pairs’. For three of them, the model displaced the positive object; for the other three, the model displaced the negative object. This allowed us to compare learning from observed successes to learning from observed errors. At the end of the model’s demonstration, three additional pairs were inserted in the list (the ‘individual pairs’) and this complete list was presented to the observer 10 times. Performance was thus evaluated over 10 hands-on trials for each pair, whether ‘social’ or ‘individual’, i.e. preceded or not by observation of a model. The order of the nine pairs composing each list never changed, only the left/right position of the positive object was pseudo-randomized across the repetitions of the list.

When the demonstration was performed by a monkey model, a reward was concealed under both objects to secure modeling of the correct choice, whereas neither well was baited to secure modeling of the erroneous choice. The same trick was used to balance, over the course of the experiment, the number of positive and negative outcomes experienced by the animals on their first encounter with ‘individual’ pairs. This way, individual learning scores, which served as a baseline to assess the models’ effect, contained a strict 50/50 mix of successes and errors on trial 1.

### Models

Three models were successively tested, in the same sequence for all six monkeys. Because we showed earlier that macaques drew the same benefit from observation whether the model made 2, 4 or 10 successive demonstrations [8], we chose here to simply provide for each model as many demonstrations as the observer would tolerate, or the model cooperate with.

#### Monkey model

The first model, the monkey model, was one of the observer’s housemates, each monkey being tested with the partner he/she was the most willing to work with. A single demonstration of the six ‘social’ pairs was provided as the monkey model could be tricked into making either a correct response or an error only once. Each animal underwent 10 different sessions (i.e. 10 different 9-pair lists) with the monkey model. A subset of the data collected with this model (the percent correct responses on the second encounter with a pair) was reported previously [10].

#### ‘Stimulus-enhancing’ human model

The second model, the ‘stimulus-enhancing’ model, was selected among four female experimenters depending on their availability. As in Meunier et al. [8], this human model captured the observer’s attention, e.g. by pushing the tray halfway towards the observer. Once sure that the animal was looking at the tray, the model displaced one of the two objects without consuming the reward if one was uncovered. As this model entailed systematic thwarting of the animal’s attempts to reach for the objects and rewards, we limited the demonstration to two successive presentations of the six ‘social’ pairs. The model always displaced the negative object for three pairs and always the positive object for the other three. Each monkey underwent 3 different sessions and as many 9-pair lists with the ‘stimulus-enhancing’ model.

#### Human model

The third model was a human selected among the same four female experimenters. This ‘monkey-like’ human was intended to mimic as closely as possible the conspecific model. The model always kept the tray out of the animal’s reach and made no effort to capture the observer’s attention, relying instead on the animals’ spontaneous willingness to observe social partners. She simply displaced one of the two objects and ate the candy if one was uncovered (care was taken to keep the tray, objects, and candies used by this model out of the animals’ contact). As the ‘monkey-like’ model left the animal free to observe or not, this model made four consecutive demonstrations of the six ‘social’ pairs, showing only errors for three pairs and only successes for the other three. Each monkey underwent 8 different sessions and as many lists with the ‘monkey-like’ human model.

For the male trio, each animal was tested with at least 2 different experimenters. At least one of them successively acting as ‘stimulus-enhancing’ and ‘monkey-like’; the other(s) intervened solely in the ‘monkey-like’ role. The ‘monkey-like’ model was found to be equally efficient whether or not it had appeared before in the ‘stimulus-enhancing’ role. So, the female trio was subsequently tested with a single female experimenter successively endorsing the ‘stimulus-enhancing’ and ‘monkey-like’ roles. Note that the two human models differed the most when showing a success (one sought the animal’s attention, the other not, and one neglected earned food treats, while the other consumed them). When showing an error, their behavior was more similar as both displaced an object and uncovered an empty food well.

### Data Collection and Analyses

#### Overall learning Δs

Raw scores were the number of errors committed over the 10 hands-on trials the animals executed for each pair, whether ‘individual’ or ‘social’. Learning Δs (individual score – social score/individual score * 100) were calculated to quantify each model’s overall influence, regardless of the outcome of the first encounter with a pair. A positive learning Δ denotes fewer errors for ‘social’ pairs than for the ‘individual’ pairs tested during the very same sessions, i.e. a beneficial model. A negative learning Δ denotes more errors for ‘social’ than for ‘individual’ pairs, i.e. a detrimental model. Note that, for overall learning Δs, both social and individual scores comprised, by design, an equal mix of successes and errors on trial 1.

#### Learning from observed successes vs observed errors

Because we showed earlier that observed errors and observed successes are not equipotential and that social learning is most helpful when monkeys (and humans) are required to learn from errors [10], we analyzed the impact of the outcome of the model’s demonstration. We calculated separate learning Δs for the ‘social’ pairs for which the model’s demonstrated the correct response and for the ‘social’ pairs for which the model’s demonstrated the incorrect response. We used the same formula as above (individual score – social score/individual score * 100) and the same individual scores. Hence, this time, learning Δs compared social scores with only successes or only errors on trial 1 to individual scores observed during the same sessions with a 50/50 mix of successes and errors on trial 1.

#### Statistics

The models’ influence on learning Δs was assessed using the SYSTAT statistical software (Version 13 for Microsoft Windows). One-sample *t*-tests were performed to determine whether learning Δ*s* significantly differed from zero, i.e. whether the model’s demonstration significantly altered subsequent learning. Parametric ANOVAs with the Huynh-Feldt adjustment (^Huynh-Feldt^p) for repeated measures followed by pairwise comparisons were used to compare the three models and paired t-tests to compare only the two human models. ANOVAs included one-way ANOVAs with the learning condition (social/individual) as the sole factor, and two-way ANOVAs with the learning condition and the first exposure’s outcome (error/success) as factors. Note that carrying a non-parametric analysis, as often recommended for small samples (see e.g. http://www.anastats.fr/index.htm), using one-sample Wilcoxon Signed-Rank Tests and Quade tests followed by pairwise comparisons, led to the same conclusions as those described below after parametric tests.

## Results

**Figure 2:**
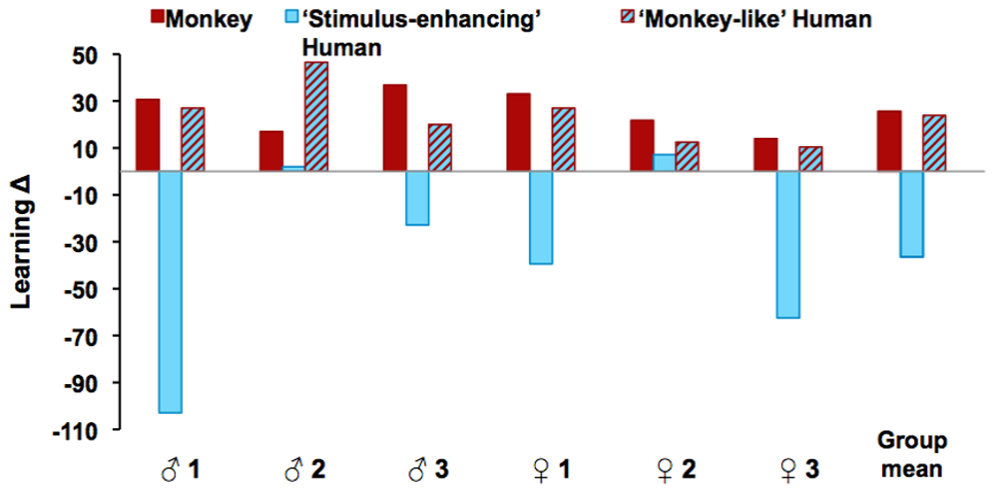
Effectiveness of the monkey model and of the two human models (‘stimulus-enhancing’ vs. ‘monkey-like’). A positive learning Δ denotes fewer errors for ‘social’ pairs than for the ‘individual’ pairs tested during the very same sessions, i.e. a beneficial model. A negative learning Δ denotes more errors for ‘social’ than for ‘individual’ pairs, i.e. a detrimental model. Results are illustrated for each monkey and for the group. Monkeys are grouped per trio of housemates according to their rank in the group hierarchy. For the detrimental ‘stimulus-enhancing’ model, two bars were truncated to keep the figure balanced. The actual scores were −103 for the top-ranking male (♂ 1) and −63 for the bottom-ranking female (♀ 3). Note that although 5/6 monkeys benefited slightly more from the monkey than from the ‘monkey-like’ human, the reverse pattern did occur too (♂ 2), hence, the indistinguishable group means yielded by the two beneficial models.

**Figure 3:**
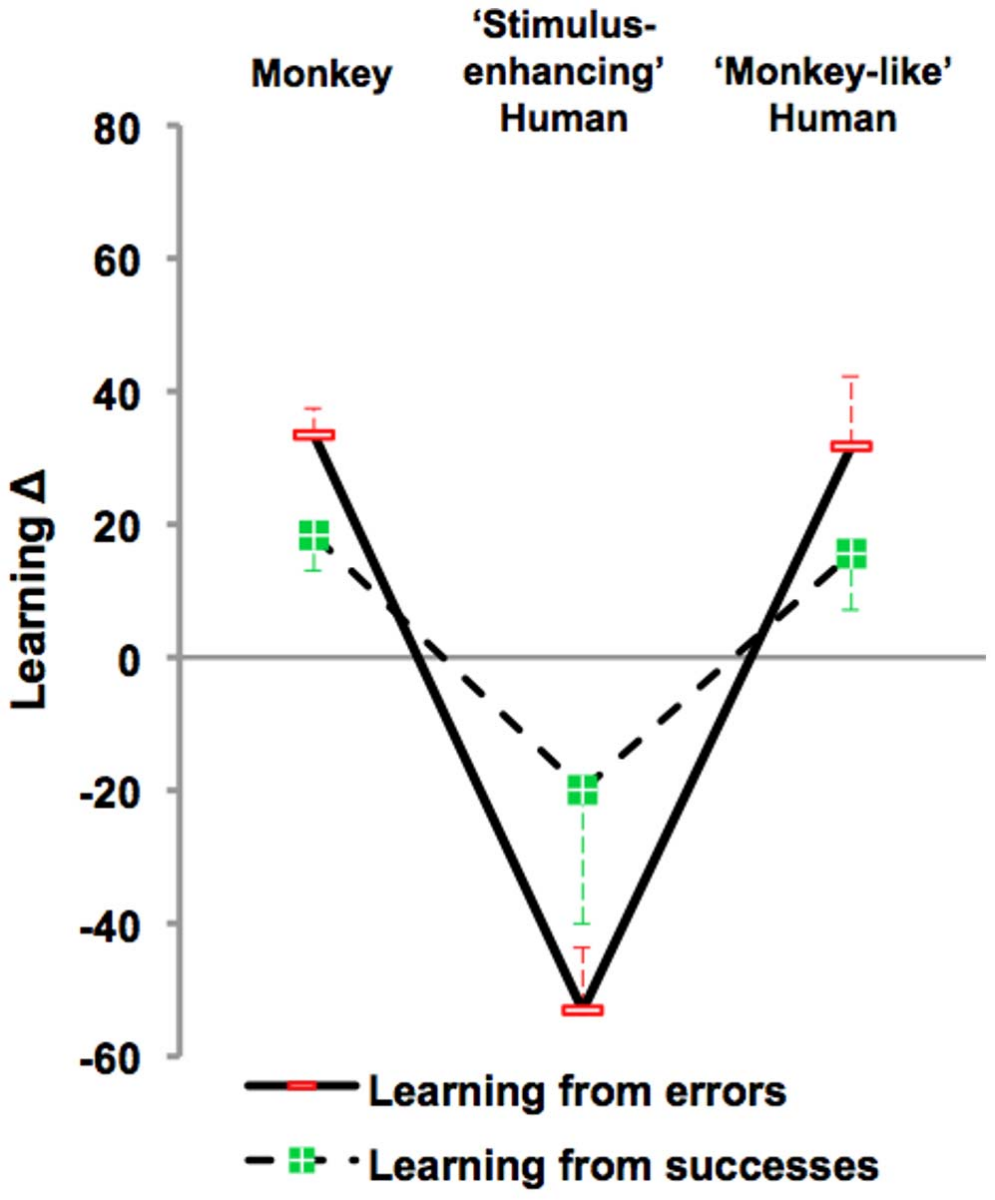
Learning from the three models’ successes vs. errors. Learning Δs were calculated separately for the ‘social’ pairs for which the model’s demonstrated the correct response and for the ‘social’ pairs for which the model’s demonstrated the incorrect response relative in both cases to scores for the ‘individual’ pairs tested during the same sessions. Group scores (mean + or – sem) are shown for each model. Note that errors widened the gap between the two effective and the ineffective models.

Figure 2 presents overall learning Δs for each monkey and for the group. Figure 3 present the group average and Table 1 the individual learning Δs calculated separately for successes and errors.

**Table 1:** Learning Δs per subject and per model calculated separately for observed successes vs. errors.

	Learning from Successes Δs			Learning from Errors Δs		
Case	Monkey	‘Stimulus-enhancing’human	‘Monkey-like’human	Monkey	‘Stimulus-enhancing’human	‘Monkey-like’human
♂ 1	34	−116	0	54	−89	29
♂ 2	−3	4	30	62	0	35
♂ 3	22	15	12	28	−59	52
**♀** 1	27	−13	50	4	−61	39
**♀** 2	19	13	−6	28	0	27
**♀** 3	11	−23	7	15	−109	19
**mean**	**18**	−**20**	**16**	**32**	−**53**	**33**
*sem*	*5*	*20*	*8*	*9*	*18*	*5*

Each learning Δ represents the gain or loss observed in the number of errors committed over 10 hands-on trials for pairs preceded by observation of a model vs. pairs learned purely individually (individual score – social score/individual score *100). Positive Δs indicate that individual learning after observation of a model was better (i.e. accompanied by less errors) than purely individual learning, whereas negative Δs correspond to a loss of performance after observation, i.e. more errors.

### Overall Effect of the Three Models

Each of the six monkeys benefited from observing one of their housemate. The gain ranged from +14 to +37%, averaging +26% for the group. Each monkey also benefited from the ‘monkey-like’ human. There, the gain ranged from +10 to +47%, averaging +24% for the group. Both changes were significant (*t*_5_ = 6.7, p = 0.001 and *t*_5_ = 4.4, p = 0.003, relative to zero, respectively).

The ‘stimulus-enhancing’ human was, on the opposite, detrimental to subsequent trial-and-error learning, yielding an average loss of performance of −37% (range +7 to −103%) that reached statistical significance (*t*_5_ = −2.1, p = 0.04). The ANOVA confirmed the difference across models (*F*_2,10_ = 11.4, ^Huynh-Feldt^p = 0.009) and the pairwise comparisons confirmed that the monkey and ‘monkey-like’ models did not differ from each other (p = 0.81), while each differing from the ‘stimulus-enhancing’ model (both p’s = 0.02).

### Learning from a Model’s Successes

When the demonstration consisted of showing the correct response, the mean group changes were modest (Figure 2), and differences across models were shallow (model effect: *F*_2,10_ = 2.6, ^Huynh-Feldt^p = 0.14). Observing another monkey making a correct choice yielded an average benefit of +18% (*t*_5_ = 3.5, p = 0.009 relative to zero). The ‘monkey-like’ human brought a comparable +16% gain (*t*_5_ = −2.1, p = 0.06). The ‘stimulus-enhancing’ human tended, on the opposite, to retard learning, yielding an average loss of −20% (*t*_5_ = −1.0, p = 0.18).

The modesty of the changes yielded by successes was accompanied by high inter-individual variability (Table 1). First, the preference of each monkey for one or the other of the effective models varied across individuals: four monkeys learned only or preferentially from a conspecific while the other two (the middle-ranking male and top-ranking female) learned only or preferentially from the ‘monkey-like’ human. Second, the animal’s reactions to the ineffective ‘stimulus-enhancing’ human’ covered a very wide spectrum, ranging from a +15% gain to a −116% loss.

### Learning from a Model’s Errors

When the demonstration consisted of showing the incorrect response, the mean group changes became substantial (Figure 2), and difference across models deepened (model effect: *F*_2,10_ = 19.9, ^Huynh-Feldt^p<0.001). The monkey model yielded a +33% gain of performance relative to purely individual learning (*t*_5_ = 7.2, p<0.001 relative to zero). The ‘monkey-like’ human brought a similar +32% gain (*t*_5_ = 3.4, p = 0.009). The ‘stimulus-enhancing’ human resulted, on the opposite, in a loss of performance averaging −53% (*t*_5_ = −2.9, p = 0.02). Pairwise comparisons confirmed that the monkey and ‘monkey-like’ models did not differ from each other (p = 0.87), while each markedly differed from the ‘stimulus-enhancing’ human (both p’s = 0.005).

The changes yielded by observed errors were also remarkably reliable across animals (Table 1). All six animals, without exception, slightly to substantially benefited from both the monkey and ‘monkey-like’ models. Not a single animal drew the slightest benefit from the ’stimulus-enhancing’ human, the effect was null at best, but in the majority of cases (4/6), the animals were perturbed as if unduly repeating the model’s errors instead of avoiding them.

### Modeled Errors vs. Successes

To sum up, showing errors rather than successes maximized the models’ influence, rendering the monkey and ‘monkey-like’ models optimal, while aggravating the disruptive effect of the ‘stimulus-enhancing’ model (Figure 3). This was confirmed by the significant interaction yielded by a global, 3×2, model × error/success ANOVA (*F*_2,10_ = 5.3, ^Huynh-Feldt^p = 0.03).

Direct comparison of the human models using paired t-tests confirmed that the two human models had statistically indistinguishable consequences (+16% *vs.* −20%; *t*_5_ = −1.8, p = 0.13) when their behavior differed the most, i.e. when showing successes, whereas they had radically opposite consequences (+32% *vs* −53; *t*_5_ = 4.8, p = 0.005) when their behavior differed the least, i.e. when showing errors. This indicates that the observer’s subjective perception of the model superseded objective differences in behavior to determine the model’s effectiveness.

## Discussion

The present study used an object discrimination task to determine what make monkeys learn from humans. We show that, to be successful, a human model has to demonstrate a behavior that resembles the monkey’s own. Specifically, a ‘stimulus-enhancing’ human actively drawing the animal’s attention to either the rewarded or the unrewarded object, but not actually performing the task, was of little help to the animals and tended, on the opposite, to perturb them. In the same animals, a human model who simply performed the task and relied on monkeys’ spontaneous tendency to observe others, facilitated learning as much as a conspecific did. This identifies model-observer similarity in behavior as a social learning mediator in rhesus macaques whose absence precludes any transmission of knowledge. By comparison, modeling errors rather than successes had a mere moderator role. Errors rendered the helpful models more beneficial, and the disruptive one more detrimental, but did not suffice, per se, to turn an ineffective model into an effective one.

### Model’s Errors: a Moderator of Social Learning

Human and non-human animals, including monkeys, can learn from other’s successes [7], [10], [12], [13], but they learn most effectively from others’ errors [10], [38], [42]. As emphasized earlier [10], this likely results from choice-induced preference, a cognitive bias shared by humans [43], [44] and monkeys, whether capuchins [45], [46], or macaques [47]. Subjects value an option more when they select it, regardless of its outcome. This preference does not operate when subjects see others select an option. Hence, others’ errors are much easier to correct than personal ones. Accordingly, although they do share the same neural processes as personal errors (error/feedback-related negativity [48]–[50]), others’ errors nevertheless have their own neural signature. Human fMRI showed that several cortical regions are uniquely activated by observed errors [48], [51] while monkey recordings revealed a subset of cells in the monkey medial frontal cortex that specifically encode other’s errors [52].

Earlier [10], we demonstrated that single-trial learning was better when monkeys observed one error committed by a conspecific than when they made the same error themselves. The present study extends these previous findings by showing that the benefit brought by observed errors is remarkably robust as 1) it persists even after 10 hands-on trials and 2) it operates even when errors are made by a heterospecific model. Modeling errors therefore appears as a powerful moderator of social learning. It could be especially useful to optimize models in future studies.

### Model-observer Similarity in Behavior: a Mediator of Social Learning

As already evoked in the Introduction, similarity in many attributes including gender, age, general background, level of competence, kinship, social status, temperament, etc. promotes social transmission of knowledge among conspecifics in human and non-human primates [27], [31]. The present study adds a new variable to the list, namely, similarity in behavior between model and observer. We showed that this was the critical factor for rhesus macaques to learn from a heterospecific model. This solves the apparent contradiction among earlier studies reporting ineffective [8], [12] vs. effective [15]–[17] human-to-monkey transmission of reward-based skills.

Similarity, actual or perceived, promotes social learning but also breeds attraction and fosters bonding [53], [54]. We feel attracted to people merely because their taste in music mirrors our own [55] and to music merely because the people that like it resemble us [56]. The similarity-breeds-attraction principle holds for non-human primates as well. In rhesus monkeys, juveniles maintain long-lasting friendships with peers whose temperament resembles their own [57] and adult females establish bonds with females whom they most resemble in age, background and status [58]. Bonding could therefore be the missing link by which similarity exerts its influence on social learning [59]. Social closeness and affiliation indeed predict transmission of knowledge among apes [28] and monkeys [60].

Here, monkeys may have failed to learn from the ‘stimulus-enhancing’ human because they could not identify to and bond with a model whose behavior (neglecting high-value food) made no sense to them. Remarkably, the two present human models had opposite consequences when their actual behavior (showing an unrewarded choice) was the same. This reinforces the idea that what made knowledge transmission succeed was the monkeys’ subjective perception of the model, not the model’s actual behavior.

### Monkeys May Need to Perceive a ‘Like-me-ness’ in the Model as do Preverbal Infants

Dissimilarity makes it difficult for scientists to form and maintain inter-disciplinary collaborative ties [61]. So, belonging to a different species should be an insurmountable dissimilarity preventing any bonding and any learning. Yet, showing monkeys a behavior that made sense to them was enough to overrule the cross-species gap and the obvious breach in similarity it represents. Why? The mechanism at play here is probably the same as that described in preverbal infant confronted with non-human agents. Infants do follow the gaze of a robot [20] and they can learn from a puppet [21] if they perceive the puppet or the robot as having a behavior that resembles their own [22]. The ‘like-me-ness’ concept may therefore provide a useful interpretive framework to explain the way monkeys and humans determine who/what to bond with and learn from. It can also help refined methods used to train laboratory monkeys involved in neuroscience studies, but also educational methods used to teach normal and disabled children.

### Alternative Learning Mechanisms

In the mechanistic view of learning, it has been suggested that much of what passes for observational learning can be explained by ‘simpler’ mechanisms such as social facilitation, stimulus enhancement, or vicarious reinforcement (see e.g. [62], [63] for reviews). The first two alternatives can be safely ruled out here. Social facilitation is the positive effect of the sheer presence of others [64]. It is irrelevant here because, whatever the model, monkeys were always tested in presence of their habitual companions. Attentional mechanisms akin to stimulus enhancement [19] can likewise be excluded as drawing attention to the pairing of an object with a reward, as did the ‘stimulus-enhancing’ model, was not sufficient to ease learning. The two human models differed in the number of demonstrations they made and in whether or not they actively drew the observer’s attention. Did the ‘stimulus-enhancing’ model fail because it made only two demonstrations compared to four for the ‘monkey-like’ model? This seems unlikely. First, as the monkey model shows here, monkeys can learn from a single demonstration. Second, we varied earlier the number of demonstrations (by a monkey model) from 2 to 10 in the same paradigm [8] and found no significant effect of the demonstration length on the benefit brought by observation. Did the ‘stimulus-enhancing’ model fail solely because it actively attracted the observer’s attention? This also seems unlikely as Brosnan and de Waal reported the same failure with a ‘stimulus-enhancing’ human that made no special attempt to attract the observer’s attention [12, see also 13].

What the present and earlier [17] results make clear is the importance of reward consumption for successful human-to-monkey transmission. Reward consumption may help simply by providing vicarious reinforcement, the processing of others’ gains known to influence decision in monkeys [15], [65], [66]. It may operate as a mere resonance mechanism automatically creating stimulus-outcome associations, but we do not think so for two reasons. First, if vicarious experience automatically induced learning, then the present animals should have learned from the ‘stimulus-enhancing’ model’s errors. Second, monkeys can copy without ever seeing another receive rewards [14], [67], so vicarious reinforcement likely moderates rather than mediates social learning. We propose instead that reward consumption ensures human-to-monkey knowledge transmission because it creates the “like-me-ness” between the observer and the model that ensures the identification and bonding necessary for knowledge transmission.
